# Does birth weight affect neonatal body weight, growth, and physiology in an animal model?

**DOI:** 10.1371/journal.pone.0246954

**Published:** 2021-02-16

**Authors:** Khaled Adjerid, Christopher J. Mayerl, Francois D. H. Gould, Chloe E. Edmonds, Bethany M. Stricklen, Laura E. Bond, Rebecca Z. German

**Affiliations:** 1 Department of Anatomy and Neurobiology, Northeast Ohio Medical University, Rootstown, Ohio, United States of America; 2 Department of Cell Biology and Neuroscience, Rowan University School of Osteopathic Medicine, Stratford, New Jersey, United States of America; University of Illinois, UNITED STATES

## Abstract

Infant birth weight affects neuromotor and biomechanical swallowing performance in infant pig models. Preterm infants are generally born low birth weight and suffer from delayed development and neuromotor deficits. These deficits include critical life skills such as swallowing and breathing. It is unclear whether these neuromotor and biomechanical deficits are a result of low birth weight or preterm birth. In this study we ask: are preterm infants simply low birth weight infants or do preterm infants differ from term infants in weight gain and swallowing behaviors independent of birth weight? We use a validated infant pig model to show that preterm and term infants gain weight differently and that birth weight is not a strong predictor of functional deficits in preterm infant swallowing. We found that preterm infants gained weight at a faster rate than term infants and with nearly three times the variation. Additionally, we found that the number of sucks per swallow, swallow duration, and the delay of the swallows relative to the suck cycles were not impacted by birth weight. These results suggest that any correlation of developmental or swallowing deficits with reduced birth weight are likely linked to underlying physiological immaturity of the preterm infant.

## Introduction

Birth weight is an important predictor of neuromotor and physiological performance in early perinatal development of mammals [1–4]. Our knowledge of the impact of birth weight on growth and performance falls into two broad categories of research. First, studies have examined the relationship between birth weight and locomotor performance of neuromuscular systems within the first days of postnatal development. For example, Vanden Hole and colleagues specifically focused on detailed relationships of muscular development and force generation as a function of birth weight using newborn infant pigs [1, 3]. They found that lower birth weight in these mammalian infant resulted in a reduced chemical, neuromuscular, and biomechanical ability to generate force for locomotion [1–4]. However, the longitudinal effects of how low birth weight may impact systems outside of locomotion, including those related to feeding, is unknown.

The second category of infant birth weight studies is more clinical in nature. The questions posed in these studies encompass a wider range of physiologic processes, including long-term growth and feeding behavior [5, 6]. These studies had a tendency to analyze the effects of low birth weight, many of which may have been a result preterm birth [5, 6]. Broad longitudinal data from both term and preterm human infants indicated that there are long-term effects of low birth weight such as reduced growth potentials and propensity for chronic disorders and obesity [5, 7–14]. In the case of preterm infant pigs serving as human infant models, there was reduced coordination in behaviors related to sucking, swallowing, and respiration relative to their term counterparts [6, 15–21].

Preterm infants are born at lower weights than their term counterparts and experience lowered growth potentials into adulthood [7, 9]. Although there is extensive literature illustrating that preterm infants struggle on issues of neural control and maturation, most clinical research studying the impact of birth weight on performance fails to account for this, and treats low birth weight preterm infants similarly to term infants [5, 7]. Prematurity is accompanied by an array of problems that small body size does not fully account for [16, 22–25]. In particular, Barlow and colleagues point out that “For the premature infant, extrauterine life is a pathological condition, which greatly amplifies the challenges to the brain in establishing functional oromotor behaviors.” Thus functional deficits in preterm infants could be the result of decreased size, a shortened period of intrauterine development, or a combination of the two [26].

In this study, we ask: are preterm infants simply low birth weight infants, or are their rate of growth and neurological development distinct from those of term infants? Using a validated infant pig model [27], we hypothesize that:

1. While preterm infant pigs will start out at lower birth weight, they will gain weight at the same rate as the term infant pigs. Alternatively, preterm infants may gain weight at either a lower or higher rate than their term counterparts.
2. Birth weight will explain some variation and differences in physiological swallowing behavior, but birth status (term vs. preterm) will be more of a determining factor. Alternatively, birth weight may be the dominant factor that explains difference in behaviors between terms and preterm infants.

We tested these hypotheses by measuring birth weight, daily weight gain, and physiological swallowing measures in term and preterm infant pigs.

## Materials and methods

All animal experiments described in this manuscript were approved by the Northeast Ohio Medical University (NEOMED) Institutional Animal Care and Use Committee protocol #17-04-071. The data were collected between 2017–2019, and used for several published studies [6, 28–30]. While birth weight was collected for all individuals, none of the previous studies included an in-depth analysis of birth weight exclusively. Details on materials and methods can be found in these papers and are briefly reviewed here.

### Animals and procedures

This study used a total of 25 piglets (*Sus scrofa)* from 6 Yorkshire/Landrace cross sows (Shoup Investments Ltd, Orrville, OH) either at term, or at seven days preterm. Preterm neonatal pigs were delivered at 108 days gestation via cesarean section, equivalent to approximately 30 weeks human gestation [27]. To eliminate any effect of vaginal birth on feeding performance, full term pigs were delivered via cesarean section at 114 days. A detailed description of cesarean delivery can be found in previous studies [19, 31, 32]. Infant pigs were weighed at birth and initially bottle-fed colostrum replacer (CL Sow Replacer, Cuprem Inc, Kenesaw, NE) within their first few hours. They were then weaned onto a daily regimen of bottle-fed milk replacement formula by 24 hours (SoluStart, Land O’ Lakes, Arden Mills, MN) for the remaining duration of the experiment, following previously developed standard protocols for infant pig care [6, 19, 30]. Body weight was measured daily, and piglets were limit-fed volume per body weight (preterm: 150 ml/kg daily–term: 300 ml/kg daily) to prevent overfeeding and related complications during the first 5–7 days of life. Feed times spacing was such that the pigs received adequate amounts of milk and time to digest. The spacing between feeds began at 3 hours for preterm infants and 4 hours for term infants and increased to 8 hours as the pigs increased in age from birth to day 17.

### Videofluoroscopy protocol and data processing

Physiologic data included in these analyses were taken from previous studies whereby videofluoroscopic data was recorded at seven days (roughly equivalent to 1–2 months post-natal in humans) and 17 days postnatal (roughly equivalent to 6–9 months of human development) [6, 29, 30]. At postnatal day seven, the preterm infant pigs were capable of maintaining their body temperature outside of animal care facilities and could feed safely during recordings. Postnatal day 17 was chosen in previous as an approximate point at which weaning could occur [6, 29, 30]. Physiological measures were identified in videofluoroscopic swallow study (VFSS) recordings as described in previous work [6]. Behavioral and physiologic parameters extracted from these data included duration of swallows, suck-swallow delay, and sucks per swallow. Swallows durations was defined as beginning when a milk bolus aggregated in the supraglottic space prior to passing beyond the epiglottis and ending when epiglottis returns to its original position and the milk has cleared the supraglottic space [28]. Suck-swallow delay was identified as the percentage of the suck cycle in which the swallow occurred. Sucks per swallow was defined as the number of sucks that occurred after a swallow and prior to the next suck accompanied by a swallow [6].

### Data organization

The first hypothesis tested differences in weight gains between term and preterm infants. To address this hypothesis, we analyzed weight data from seven preterm and five term infants from two sows (first analyzed in Mayerl et al., 2020a, b) in two ways [29, 30]. We first analyzed weight gain using chronological age, the number of days past birth for both term and preterm infants [33]. We then analyzed weight data for corrected age, the chronological age minus the number of days preterm, or days born prior to full gestation. Thus, our preterm infants, delivered seven days prior to term, would be age zero at seven days post-birth. Additionally, when analyzing weight gain, we used both raw weight and weight scaled to birth weight. To test whether weight gain through the first 17 days of life differed between preterm and term infants, we made three comparisons: (1) chronological age/raw weight (2) chronological age/birth weight-scaled weight; (3) corrected age/raw weight. To test the second hypothesis, we used data from 10 preterm, and 11 term infants (first used for Mayerl et al., 2019 and 2020 a,b) at chronological ages seven and 17 days [6, 29, 30]. Because this is a retrospective study, it is limited in the number of litters and individuals that were available for this analysis. A comprehensive list of specimens, sows, and sample sizes is included in Table 1.

**Table 1:** Sample sizes of infants from each litter used in weight gain analysis and swallow performance analysis.

	PT1:	PT2:	PT3:	T1:	T2:	T3:	Sample Size
	Sow 1	Sow 2	Sow 3	Sow 4	Sow 5	Sow 6	
Term Status	Preterm	Preterm	Preterm	Term	Term	Term	
Weight Gain	5			7			N = 12
Swallow Performance	3	1	4	5	4	4	N = 21
							Total N = 25 (12 PT + 13 T)

Sample sizes of the specimens used in the weight gain and swallow performance studies. In the weight gain study, we used 5 preterm and 7 term piglets from 2 sows. In the swallow performance study, we used 8 preterm piglets from 3 sows (including 3 preterm piglets from the sow used in weight gain study) and 13 term piglets from 3 other sows (including 5 term piglets from the sow used in weight gain study).

### Statistical analyses

To test our first hypothesis regarding differences in weight gain, we first used paired t-tests in Excel (Microsoft Corporation, Redmond, WA) as well as generalized linear models with litter as a random effect in R Studio to compare weights of preterm and term infant litters on day one and again on day 17 [34]. Then to test differences in variation observed between preterm and term infants, we first calculated coefficient of variation for both preterm and term infants, and then we used a Levene’s test to check for significance in difference in variation on day one, seven, ten, 16 and 17 using Real Statistics Add-in in Excel [35].

To test the second hypothesis concerning the effects of birth weight on swallow performance, we conducted three individual covariate analyses using Systat (Systat Software, San Jose, CA). We first ran an ANCOVA analysis, with a single physiologic variable (swallow duration, sucks per swallow, and suck-swallow delay) as the response and with treatment group as a fixed factor with four levels: preterm day seven, term day seven, preterm day 17, term day 17. Birth weight was listed as a covariate and individual as a random factor. The interaction term tested whether the linear relationship between birth weight and physiologic variable was the same for each of the groups [36]. If there was significant interaction, then a set of subsequent regressions were carried out to determine the relationship between these two variables. If the interactions were not significant, then an additional model testing the main effect of birth weight across all treatment groups without the interaction term was run. Lastly, a similar test was run but with sex as the random factor to test any differences in the population.

Sample sizes used herein were deemed to be sufficient based on power analyses and variation in subtle behaviors observed in previous studies [37, 38]. Additionally, as these are domesticated animals, genetic variation in growth or physiological parameters between litters were considered negligible when compared to variation among individuals [39]. Thus, for this analysis, litter or sow were not considered as factors.

## Results

### Weight gain

**Fig 1:**
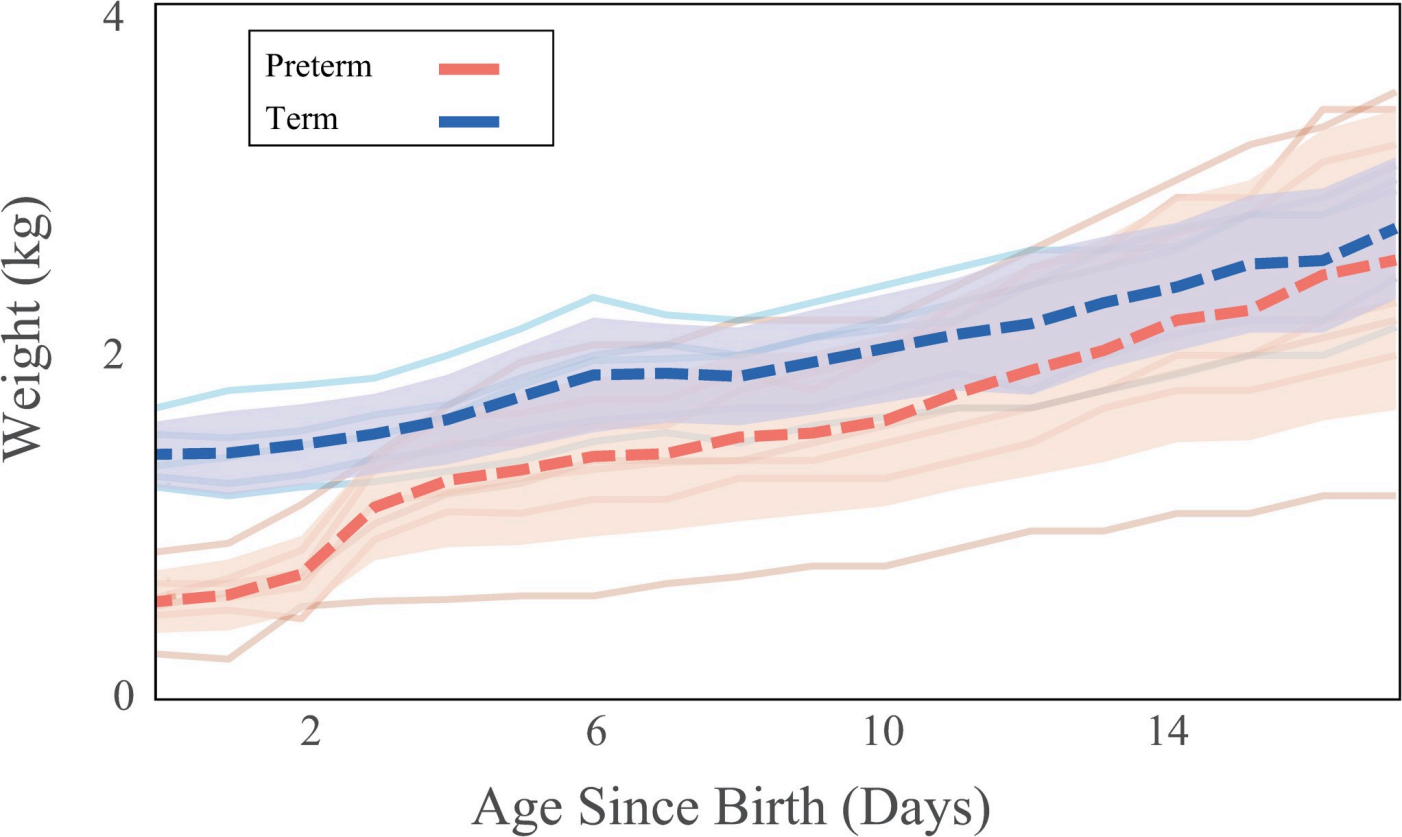
Gross weight gain trends. Gross weight gain for the postnatal period starting from birth through day 17. Dashed lines indicate mean weight and shaded regions indicate 95% confidence intervals calculated at each day. Colors indicate birth status for term (blue) and preterm infants (red).

Birth weight was lower in preterm than in term infants (PT_BW_ = 0.58 ± 0.15 kg, T_BW_ = 1.40 ± 0.16 kg, p < 0.001, t_crit_ = 2.306). At day four, preterm infants’ weight was equivalent to the terms’ and gained weight at a similar rate through day 17 (Fig 1). By day 17, there was no significant difference between preterm and term body weights (PT_D17_ = 2.56 ± 0.92 kg, T_D17_ = 2.58 ± 0.32 kg, p = 0.635, t_crit_ = 2.262). Levene’s test revealed that at days one, seven, and 10, the amount of variation between preterm and term was not significantly different (p > 0.05). However, by day 16, the variation in the preterm infants was significantly higher than in the terms (p = 0.038, f = 5.89). When using corrected age (T_D0,_ PT_D7_), birth weights were similar however, at day 17 (T_D17,_ PT_D24_) body weight was greater in preterm infants than in term infants.

**Fig 2:**
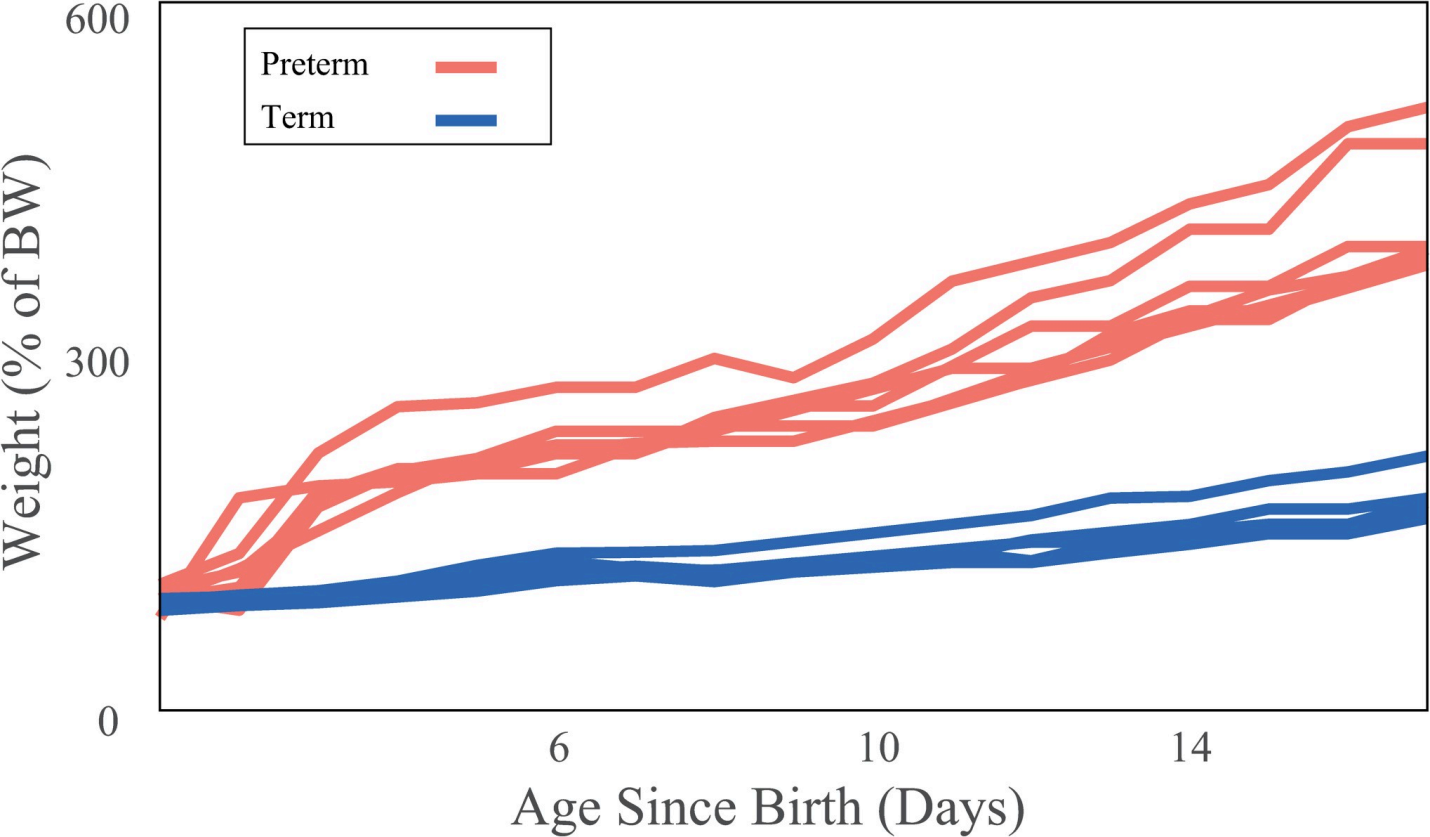
Body weight scaled weight gain trends. Percent weight gain scaled to initial birth weight during the postnatal period starting at day one through day 17. Colors indicate birth status for term (blue) and preterm infants (red).

When scaling weight gain by initial birth weight, we saw further amplified differences between preterm and term infants (Fig 2). By the end of the study (day 17), preterm infants weighed more than twice the term infants’ corrected bodyweight (W_17PT scaled_ = 422 ± 29%g, W_17T scaled_ = 190 ± 4%, p < 0.001, t_crit_ = 2.306). The daily coefficient of variation in the preterm infants was consistently higher than the term infants throughout (CoV_Taverage_ = 0.070 ±0.03, CoV_PTaverage_ = 0.111±0.04, p < 0.001) and Levene’s test revealed that by day 16, the variation was also greater in preterm infants that in terms (p = 0.05, f = 4.91).

**Fig 3:**
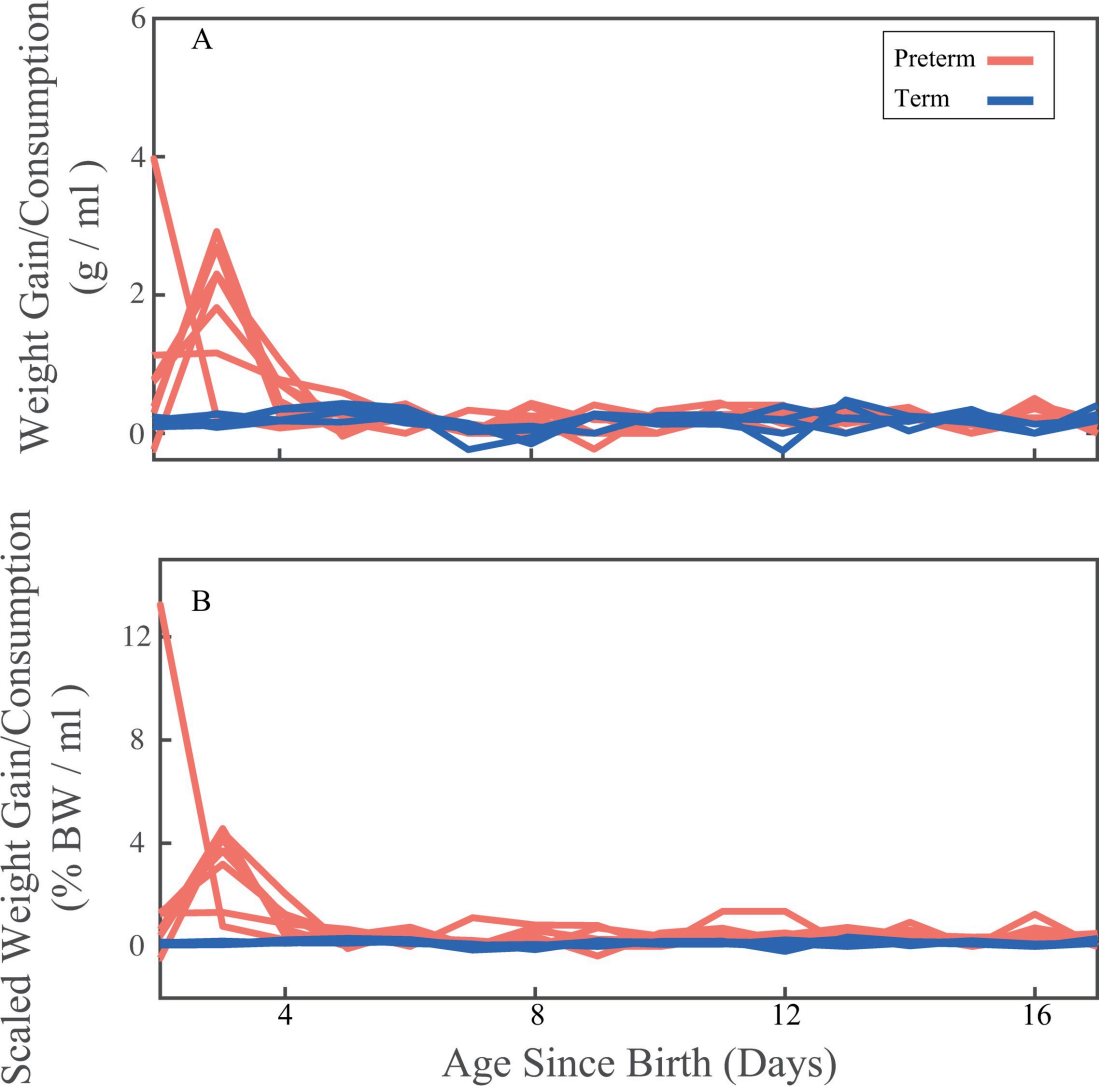
Consumption corrected weight gain trends. Daily weight gain during the postnatal period starting at day one through day 17 expressed as a function of consumption in g/ml (A), and birthweight scaled body weight expressed as a function of consumption expressed body weight as percentage of birthweight / ml consumed (B). Colors indicate birth status for term (blue) and preterm infants (red).

When measuring the mean daily weight gained as a function of consumption (Fig 3A, expressed as gram weight gained/ml milk consumed), we found that the preterm infants gained more daily weight relative to their consumption than term infants (average daily WG_PT_ = 0.37 ± 0.46 g/ml, WG_T_ = 0.17 ± 0.10 g/ml, p = 0.05, t_crit_ = 2.1). When scaling the weight gain by their initial birth weight (Fig 3B expressed as birthweight scaled weight gained/ml milk consumed), we see an even more pronounced difference in weight gain trends between the preterm infants and the term infants (average daily WG_PT scaled_ = 0.68 ± 0.85 kg, WG_T scaled_ = 0.12 ± 0.07 kg, p < 0.05, t_crit_ = 2.12). The weight gained in first 5 days since birth (daily WG_PT d1-d5_ = 0.79 ± 0.81 kg, WG_T d1-d5_ = 0.20 ± 0.05 kg) was higher than in the remaining postnatal days (daily WG_PT d6-d17_ = 0.19 ± 0.09 kg vs WG_T d6-d17_ = 0.15 ± 0.10 kg) where the weight gain was similar.

### Physiologic behaviors

**Fig 4:**
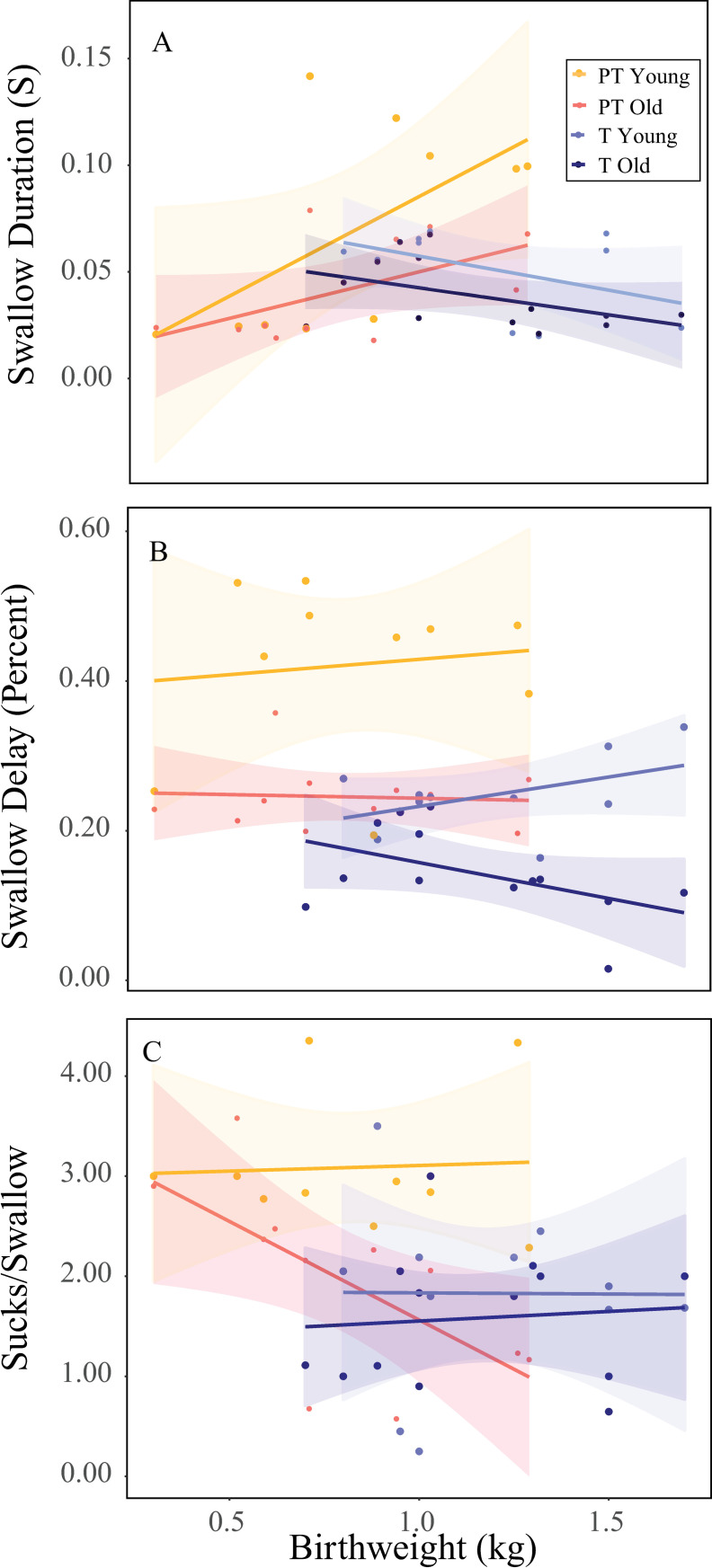
Swallowing behaviors vs. birth weight. Swallow duration in seconds, swallow delay as percentage of total swallow duration, and sucks per swallow plotted vs. birth weight by birth status (term vs. preterm) and neonatal age (young/seven days and old/17 days). Individual colors indicate the different treatment combinations of term status and age. Lines indicate linear regressions and shaded regions indicate 95% confidence intervals.

For swallow duration (Fig 4A), the ANCOVA (Table 2) indicated that there was a significant interaction (p < 0.05) between birth weight and the treatment groups (preterm/day seven, term/day seven, preterm/day 17, term /day 17). Subsequent regressions found no relationship between swallow duration and birth weight in any treatment group (Table 3). For sucks per swallow and suck-swallow delay (Fig 4B and 4C), there was no significant interaction, indicating that no group had a different relationship between sucks per swallow and birth weight, or between swallow delay and birth weight. The subsequent model indicated no overall relationship with birth weight, although there were differences among the treatment groups in both sucks per swallow and suck-swallow delay consistent with the results found in the previous study analyzing these swallowing behaviors independent of birth weight [6]. Sex was not found to be a factor in any of the physiologic behaviors (P > 0.05).

**Table 2:** Statistical analysis results for the physiological swallowing measures.

Measure		F-statistic	P-value	Random individual variance/Error variance
Swallow Duration				0.0004/0.0002
	Birth weight	2.3799	0.1314	
	Treatment	2.9464	0.0454	
	Interaction Birth weight*Treatment	5.2768	0.0039	
Swallow Delay				0.0000/0.0051
	Birth weight	0.0421	0.8399	
	Treatment	9.5979	0.0006	
	Interaction Birth weight*Treatment	1.1208	0.3684	
Sucks/Swallow				0.0000/0.6167
	Birth weight	2.0120	0.1741	
	Treatment	0.8897	0.4664	
	Interaction Birth weight*Treatment	1.6722	0.2106	

Statistics for physiological swallowing measure**s** compared with the birth weight as a predictor. Resulting F-statistics, P-values, and ratios of individual as source of variance to overall error are shown for swallow duration, swallow delay, and sucks per swallow.

**Table 3:** Statistical analysis results for swallow duration individual linear regressions.

Swallow Duration		Slope	Intercept	F-statistic	P-value	R^2^
	PT- Young	0.6077	0.0924	4.6846	0.0624	0.369
	PT–Old	0.5513	0.0432	3.9297	0.0788	0.304
	Term–Young	-0.4703	-0.0316	2.5563	0.1443	0.221
	Term–Old	-0.4622	-0.0251	2.9876	0.1118	0.214

Resulting Slopes, Intercepts, P-values, F-statistics and R^2^ Statistical analysis results for individual linear regressions done for each treatment group computer for swallow duration.

## Discussion

### Weight gain

The pattern of weight gain in preterm infants was distinct from that of term infants. As hypothesized, preterm infants had lower birth weights than their term counterparts. However, the preterm infants rapidly gained weight and ‘caught up’ to the term infants. While the mean weight at 17 days was not significantly different between the two groups, the variation among the preterm infants was higher than that of the term infants. The term infants’ lower variation across the latter end of postnatal period analyzed in this study suggests that more time in utero permits for more uniform growth amongst littermates. It also suggests a larger variation in response to the preterm infants’ reduced development time.

Differences in weight gains scaled to initial birth weight were such that preterm infants gained weight at a greater rate than term infants. Term infants’ weight doubled from their birth weight while preterm infants grew to more than four times their birth weight in the same postnatal period, though interindividual variation was much greater in the preterm infants. It may be that in response to reduced uterine development and lower birth weight; preterm infants were able to ‘catch up’ to the higher birth weight individuals by gaining weight more rapidly and being more metabolically efficient with their feed than in term infants. One limitation of this measure is that the daily weights were only scaled by the first weight on day one. Therefore, if an individual’s weight gain was irregular in the first 24–48 hours but was otherwise normal for the remaining duration, then this may affect the scaled weight calculation for the entirety of the postnatal period.

Using corrected ages to account for a shortened time in utero further highlighted differences between preterm and term infants. Preterm infant weights leading up to corrected day 17 (PT_D24_) did not overlap with those of the term infants, nor did the adjustment of the time scale make the growth rates appears similar to the term infants. Correcting for the degree of prematurity did not appear to put the preterm infants on the same growth trajectory as the term infants. This suggests that infants do not develop in first few weeks of life, during what would have been the end of the gestational period, in the same way had they been carried to full term. Additionally, one limitation that should be noted is that by correcting by degree of prematurity, the results that are seen are simply according to existing ‘day 17’ (actual day 24) mass and variation trends. Therefore, by correcting, it makes sense that while preterm infant birth weight aligns with term birth weight, the corrected day 17 weight is much higher as the rate of weight gain has not been corrected or adjusted in any way. However, by eschewing the initial jump in preterm weight gain seen in the first three days, the weight differences between the smallest and largest infants were even more pronounced by age correction. It becomes even more obvious that in preterm infants, individuals that may suffer from obesity or low body weight into weaning may do so to greater degree and in greater numbers relative to the term litter which exhibits less variation relative to the mean.

It is important to note that these data had significant inter-individual variability, as we have found in other studies [38, 39]. Further, because it is based on only two litters, it is not possible to get a strong estimate of inter-litter variation.

### Physiologic swallowing behaviors

Birth weight was not a good predictor of the differences between preterm and term infants for the swallowing behaviors we measured. While these variables show significant differences between preterm and term infants, as well as between day seven and day 17, birth weight did not play a role in explaining the variation within groups or between them. The only significant covariate effect birth weight had was in swallow duration. Swallow duration increased as a function of birth weight for preterm pigs, and decreased for term pigs, although this trend was not statistically significant (Table 2). This result could be a reflection of a lack of statistical power in these analyses.

Our measures of feeding behaviors did not explain patterns of weight gain in preterm and term infants given the persistent differences in swallowing function including issues with latching and sucking, reduced bolus size, and lack of aerodigestive coordination [6, 16, 28]. Furthermore, effective feeding is a clinically strong predictor of weight gain and thriving in low birth weight infants [40, 41]. However, low birth weight by itself is not predictive of post weaning oromotor difficulties in humans [42]. Indeed much of the concern about outcomes in low birth weight infants centers around oral feeding ability [43].

### Broader biological and clinical significance of birth weight

While it is not known if pigs or boars experience preterm birth in the wild, there is evidence that non-human primates may. In the cases of chimpanzees and pigtail macaques, preterm individuals had lower survival rates as a result and reduced performance in surviving individuals [44, 45]. In the case of a study conducted on chimpanzees, 17 individuals out of 67 were naturally born preterm (<208 days) and only three were recorded as live births with the rest deemed stillbirth or not viable. The lone surviving individual who reached adulthood from that study (201 days gestation) was found to have been blind from a lack of pupil development in-utero [44]. Other mothers in this study had recurrent preterm deliveries due to perinatal death of the fetus between 25–192 days.

It is worth noting that our preterm infants were feeding in a benign environment, with individual attention, ample time, and uninterrupted access to food. A more naturalistic setting for polytocous placental mammals that must compete with siblings for milk from a sow’s nipples might have amplified the effect of variation in feeding effectiveness. Similarly, recent research into adult intensive care units has shown that strain on resources can affect outcomes for complex cases [46, 47]. Therefore, in a neonatal hospital care setting where preterm infants who struggle to feed are housed, lack of a mother’s individual attention might amplify the effects of poor feeding efficiency on weight gain. Furthermore, we looked at only one gestational age of preterm infants. Many infants that are considered in preterm studies [7, 11, 14] are significantly more preterm than those considered in this study.

Understanding birth weight’s effect on development can inform outcomes and treatments. Human infants born small for their gestational age and low birth weight preterm infants have increased by 17% from 1990–2008 while the macrosomic infants have decreased by 50% within that same period [48]. Previous studies in preterm human infants have shown oromotor deficits up to 12 months of age compared to their term counterparts [42]. However, birth weight was not predictive of oromotor difficulties lasting beyond weaning [42]. Infant birth in humans that was classified as very low birth weight (VLBW < 1500 g), a category not observed in any of our preterm or term infants, was a predictor of swallowing deficits [49]. However, VLBW and ELBW (extremely low birth weight < 1000 g) birth occur almost exclusively as a result of preterm birth and are generally related to inability to feed, causing a failure to thrive [50]. Here we show that exclusive focus on the low birth weight of preterm infants may possibly overlook critical neuromotor deficiencies that may be more determining of short-term effects and chronic feeding and health consequences.

## Supporting information

S1 Table Daily weight gain measurements for term and preterm infants. Daily measurements of body weight made for term and preterm infants. Chronological age and corrected age in days is listed for preterm infants.
(DOCX)

S2 Table Swallowing behavior measurements for term and preterm infants. Measurements of different swallowing behaviors made on both preterm and term individuals at different days of the postnatal period. Litter/Sow identification is also listed to indicate whether the litter number and term status.
(DOCX)
