# Supplementary material for: Does birth weight affect neonatal body weight, growth, and physiology in an animal model?
Source: PLoS One. 2021 Feb 16;16(2):e0246954. doi: 10.1371/journal.pone.0246954 (PMC7886147; doi:10.1371/journal.pone.0246954)
Supplement: S1 Table — Daily measurements of body weight made for term and preterm infants. Chronological age and corrected age in days is listed for preterm infants. (DOCX) [file pone.0246954.s001.docx]

**S1 Table. Daily weight gain measurements for term and preterm infants.** Daily measurements of body weight made for term and preterm infants. Chronological age and corrected age in days is listed for preterm infants.

| **Birth Status** | **Individual** | **Chronological Age** | **Weight (kg)** | **Corrected Age** |
| --- | --- | --- | --- | --- |
| Preterm | 1 | Birth | 0.52 | -7 |
| Preterm | 1 | 1 | 0.55 | -6 |
| Preterm | 1 | 2 | 0.50 | -5 |
| Preterm | 1 | 3 | 0.95 | -4 |
| Preterm | 1 | 4 | 1.11 | -3 |
| Preterm | 1 | 5 | 1.10 | -2 |
| Preterm | 1 | 6 | 1.18 | -1 |
| Preterm | 1 | 7 | 1.18 | Birth Equivalent |
| Preterm | 1 | 8 | 1.30 | 1 |
| Preterm | 1 | 9 | 1.30 | 2 |
| Preterm | 1 | 10 | 1.30 | 3 |
| Preterm | 1 | 11 | 1.40 | 4 |
| Preterm | 1 | 12 | 1.50 | 5 |
| Preterm | 1 | 13 | 1.70 | 6 |
| Preterm | 1 | 14 | 1.80 | 7 |
| Preterm | 1 | 15 | 1.80 | 8 |
| Preterm | 1 | 16 | 1.90 | 9 |
| Preterm | 1 | 17 | 2.00 | 10 |
| Preterm | 1 | 18 | 2.30 | 11 |
| Preterm | 1 | 19 | 2.40 | 12 |
| Preterm | 1 | 20 | 2.70 | 13 |
| Preterm | 1 | 21 | 2.70 | 14 |
| Preterm | 1 | 22 | 2.90 | 15 |
| Preterm | 1 | 23 | 3.00 | 16 |
| Preterm | 1 | 24 | 3.12 | 17 |
| Preterm | 2 | Birth | 0.62 | -7 |
| Preterm | 2 | 1 | 0.73 | -6 |
| Preterm | 2 | 2 | 0.89 | -5 |
| Preterm | 2 | 3 | 1.41 | -4 |
| Preterm | 2 | 4 | 1.65 | -3 |
| Preterm | 2 | 5 | 1.67 | -2 |
| Preterm | 2 | 6 | 1.75 | -1 |
| Preterm | 2 | 7 | 1.75 | Birth Equivalent |
| Preterm | 2 | 8 | 1.90 | 1 |
| Preterm | 2 | 9 | 1.80 | 2 |
| Preterm | 2 | 10 | 2.00 | 3 |
| Preterm | 2 | 11 | 2.30 | 4 |
| Preterm | 2 | 12 | 2.40 | 5 |
| Preterm | 2 | 13 | 2.50 | 6 |
| Preterm | 2 | 14 | 2.70 | 7 |
| Preterm | 2 | 15 | 2.80 | 8 |
| Preterm | 2 | 16 | 3.10 | 9 |
| Preterm | 2 | 17 | 3.20 | 10 |
| Preterm | 2 | 18 | 3.50 | 11 |
| Preterm | 2 | 19 | 4.00 | 12 |
| Preterm | 2 | 20 | 4.10 | 13 |
| Preterm | 2 | 21 | 4.40 | 14 |
| Preterm | 2 | 22 | 4.70 | 15 |
| Preterm | 2 | 23 | 5.10 | 16 |
| Preterm | 2 | 24 | 5.57 | 17 |
| Preterm | 3 | Birth | 0.70 | -7 |
| Preterm | 3 | 1 | 0.70 | -6 |
| Preterm | 3 | 2 | 0.76 | -5 |
| Preterm | 3 | 3 | 1.35 | -4 |
| Preterm | 3 | 4 | 1.50 | -3 |
| Preterm | 3 | 5 | 1.50 | -2 |
| Preterm | 3 | 6 | 1.60 | -1 |
| Preterm | 3 | 7 | 1.60 | Birth Equivalent |
| Preterm | 3 | 8 | 1.80 | 1 |
| Preterm | 3 | 9 | 1.90 | 2 |
| Preterm | 3 | 10 | 2.00 | 3 |
| Preterm | 3 | 11 | 2.20 | 4 |
| Preterm | 3 | 12 | 2.50 | 5 |
| Preterm | 3 | 13 | 2.60 | 6 |
| Preterm | 3 | 14 | 2.90 | 7 |
| Preterm | 3 | 15 | 2.90 | 8 |
| Preterm | 3 | 16 | 3.40 | 9 |
| Preterm | 3 | 17 | 3.40 | 10 |
| Preterm | 3 | 18 | 3.80 | 11 |
| Preterm | 3 | 19 | 4.60 | 12 |
| Preterm | 3 | 20 | 4.70 | 13 |
| Preterm | 3 | 21 | 4.90 | 14 |
| Preterm | 3 | 22 | 5.20 | 15 |
| Preterm | 3 | 23 | 5.60 | 16 |
| Preterm | 3 | 24 | 5.62 | 17 |
| Preterm | 4 | Birth | 0.59 | -7 |
| Preterm | 4 | 1 | 0.62 | -6 |
| Preterm | 4 | 2 | 0.68 | -5 |
| Preterm | 4 | 3 | 1.16 | -4 |
| Preterm | 4 | 4 | 1.22 | -3 |
| Preterm | 4 | 5 | 1.30 | -2 |
| Preterm | 4 | 6 | 1.35 | -1 |
| Preterm | 4 | 7 | 1.39 | Birth Equivalent |
| Preterm | 4 | 8 | 1.40 | 1 |
| Preterm | 4 | 9 | 1.40 | 2 |
| Preterm | 4 | 10 | 1.50 | 3 |
| Preterm | 4 | 11 | 1.60 | 4 |
| Preterm | 4 | 12 | 1.70 | 5 |
| Preterm | 4 | 13 | 1.80 | 6 |
| Preterm | 4 | 14 | 2.00 | 7 |
| Preterm | 4 | 15 | 2.00 | 8 |
| Preterm | 4 | 16 | 2.20 | 9 |
| Preterm | 4 | 17 | 2.30 | 10 |
| Preterm | 4 | 18 | 2.40 | 11 |
| Preterm | 4 | 19 | 2.40 | 12 |
| Preterm | 4 | 20 | 2.90 | 13 |
| Preterm | 4 | 21 | 3.10 | 14 |
| Preterm | 4 | 22 | 3.20 | 15 |
| Preterm | 4 | 23 | 3.50 | 16 |
| Preterm | 4 | 24 | 4.05 | 17 |
| Preterm | 5 | Birth | 0.57 | -7 |
| Preterm | 5 | 1 | 0.65 | -6 |
| Preterm | 5 | 2 | 0.73 | -5 |
| Preterm | 5 | 3 | 1.04 | -4 |
| Preterm | 5 | 4 | 1.21 | -3 |
| Preterm | 5 | 5 | 1.27 | -2 |
| Preterm | 5 | 6 | 1.40 | -1 |
| Preterm | 5 | 7 | 1.40 | Birth Equivalent |
| Preterm | 5 | 8 | 1.40 | 1 |
| Preterm | 5 | 9 | 1.50 | 2 |
| Preterm | 5 | 10 | 1.60 | 3 |
| Preterm | 5 | 11 | 1.70 | 4 |
| Preterm | 5 | 12 | 1.70 | 5 |
| Preterm | 5 | 13 | 1.80 | 6 |
| Preterm | 5 | 14 | 1.90 | 7 |
| Preterm | 5 | 15 | 2.00 | 8 |
| Preterm | 5 | 16 | 2.10 | 9 |
| Preterm | 5 | 17 | 2.20 | 10 |
| Preterm | 5 | 18 | 2.30 | 11 |
| Preterm | 5 | 19 | 2.50 | 12 |
| Preterm | 5 | 20 | 2.70 | 13 |
| Preterm | 5 | 21 | 2.70 | 14 |
| Preterm | 5 | 22 | 2.80 | 15 |
| Preterm | 5 | 23 | 3.00 | 16 |
| Preterm | 5 | 24 | 3.35 | 17 |
| Preterm | 6 | Birth | 0.88 | -7 |
| Preterm | 6 | 1 | 0.93 | -6 |
| Preterm | 6 | 2 | 1.15 | -5 |
| Preterm | 6 | 3 | 1.43 | -4 |
| Preterm | 6 | 4 | 1.71 | -3 |
| Preterm | 6 | 5 | 1.96 | -2 |
| Preterm | 6 | 6 | 2.06 | -1 |
| Preterm | 6 | 7 | 2.06 | Birth Equivalent |
| Preterm | 6 | 8 | 2.20 | 1 |
| Preterm | 6 | 9 | 2.20 | 2 |
| Preterm | 6 | 10 | 2.20 | 3 |
| Preterm | 6 | 11 | 2.40 | 4 |
| Preterm | 6 | 12 | 2.60 | 5 |
| Preterm | 6 | 13 | 2.80 | 6 |
| Preterm | 6 | 14 | 3.00 | 7 |
| Preterm | 6 | 15 | 3.20 | 8 |
| Preterm | 6 | 16 | 3.30 | 9 |
| Preterm | 6 | 17 | 3.50 | 10 |
| Preterm | 6 | 18 | 3.60 | 11 |
| Preterm | 6 | 19 | 4.30 | 12 |
| Preterm | 6 | 20 | 4.60 | 13 |
| Preterm | 6 | 21 | 4.90 | 14 |
| Preterm | 6 | 22 | 5.20 | 15 |
| Preterm | 6 | 23 | 5.60 | 16 |
| Preterm | 6 | 24 | 5.65 | 17 |
| Preterm | 7 | Birth | 0.30 | -7 |
| Preterm | 7 | 1 | 0.27 | -6 |
| Preterm | 7 | 2 | 0.57 | -5 |
| Preterm | 7 | 3 | 0.60 | -4 |
| Preterm | 7 | 4 | 0.61 | -3 |
| Preterm | 7 | 5 | 0.63 | -2 |
| Preterm | 7 | 6 | 0.63 | -1 |
| Preterm | 7 | 7 | 0.70 | Birth Equivalent |
| Preterm | 7 | 8 | 0.74 | 1 |
| Preterm | 7 | 9 | 0.80 | 2 |
| Preterm | 7 | 10 | 0.80 | 3 |
| Preterm | 7 | 11 | 0.90 | 4 |
| Preterm | 7 | 12 | 1.00 | 5 |
| Preterm | 7 | 13 | 1.00 | 6 |
| Preterm | 7 | 14 | 1.10 | 7 |
| Preterm | 7 | 15 | 1.10 | 8 |
| Preterm | 7 | 16 | 1.20 | 9 |
| Preterm | 7 | 17 | 1.20 | 10 |
| Preterm | 7 | 18 | 1.20 | 11 |
| Preterm | 7 | 19 | 1.60 | 12 |
| Preterm | 7 | 20 | 1.50 | 13 |
| Preterm | 7 | 21 | 1.70 | 14 |
| Preterm | 7 | 22 | 1.80 | 15 |
| Preterm | 7 | 23 | 1.85 | 16 |
| Preterm | 7 | 24 | 2.40 | 17 |
| Term | 7 | Birth | 1.25 | - |
| Term | 8 | 1 | 1.20 | - |
| Term | 8 | 2 | 1.25 | - |
| Term | 8 | 3 | 1.28 | - |
| Term | 8 | 4 | 1.34 | - |
| Term | 8 | 5 | 1.40 | - |
| Term | 8 | 6 | 1.51 | - |
| Term | 8 | 7 | 1.56 | - |
| Term | 8 | 8 | 1.50 | - |
| Term | 8 | 9 | 1.60 | - |
| Term | 8 | 10 | 1.65 | - |
| Term | 8 | 11 | 1.70 | - |
| Term | 8 | 12 | 1.70 | - |
| Term | 8 | 13 | 1.80 | - |
| Term | 8 | 14 | 1.89 | - |
| Term | 8 | 15 | 2.00 | - |
| Term | 8 | 16 | 2.00 | - |
| Term | 8 | 17 | 2.16 | - |
| Term | 9 | Birth | 1.31 | - |
| Term | 9 | 1 | 1.27 | - |
| Term | 9 | 2 | 1.32 | - |
| Term | 9 | 3 | 1.41 | - |
| Term | 9 | 4 | 1.46 | - |
| Term | 9 | 5 | 1.57 | - |
| Term | 9 | 6 | 1.63 | - |
| Term | 9 | 7 | 1.66 | - |
| Term | 9 | 8 | 1.70 | - |
| Term | 9 | 9 | 1.70 | - |
| Term | 9 | 10 | 1.80 | - |
| Term | 9 | 11 | 1.90 | - |
| Term | 9 | 12 | 1.80 | - |
| Term | 9 | 13 | 2.00 | - |
| Term | 9 | 14 | 2.12 | - |
| Term | 9 | 15 | 2.20 | - |
| Term | 9 | 16 | 2.20 | - |
| Term | 9 | 17 | 2.44 | - |
| Term | 10 | Birth | 1.55 | - |
| Term | 10 | 1 | 1.53 | - |
| Term | 10 | 2 | 1.57 | - |
| Term | 10 | 3 | 1.66 | - |
| Term | 10 | 4 | 1.72 | - |
| Term | 10 | 5 | 1.87 | - |
| Term | 10 | 6 | 2.00 | - |
| Term | 10 | 7 | 2.06 | - |
| Term | 10 | 8 | 2.00 | - |
| Term | 10 | 9 | 2.10 | - |
| Term | 10 | 10 | 2.15 | - |
| Term | 10 | 11 | 2.20 | - |
| Term | 10 | 12 | 2.40 | - |
| Term | 10 | 13 | 2.50 | - |
| Term | 10 | 14 | 2.60 | - |
| Term | 10 | 15 | 2.80 | - |
| Term | 10 | 16 | 2.80 | - |
| Term | 10 | 17 | 2.94 | - |
| Term | 11 | Birth | 1.70 | - |
| Term | 11 | 1 | 1.80 | - |
| Term | 11 | 2 | 1.83 | - |
| Term | 11 | 3 | 1.87 | - |
| Term | 11 | 4 | 2.00 | - |
| Term | 11 | 5 | 2.15 | - |
| Term | 11 | 6 | 2.33 | - |
| Term | 11 | 7 | 2.23 | - |
| Term | 11 | 8 | 2.20 | - |
| Term | 11 | 9 | 2.30 | - |
| Term | 11 | 10 | 2.40 | - |
| Term | 11 | 11 | 2.50 | - |
| Term | 11 | 12 | 2.60 | - |
| Term | 11 | 13 | 2.60 | - |
| Term | 11 | 14 | 2.72 | - |
| Term | 11 | 15 | 2.80 | - |
| Term | 11 | 16 | 2.80 | - |
| Term | 11 | 17 | 3.00 | - |
| Term | 12 | Birth | 1.37 | - |
| Term | 12 | 1 | 1.42 | - |
| Term | 12 | 2 | 1.49 | - |
| Term | 12 | 3 | 1.54 | - |
| Term | 12 | 4 | 1.65 | - |
| Term | 12 | 5 | 1.83 | - |
| Term | 12 | 6 | 1.97 | - |
| Term | 12 | 7 | 1.98 | - |
| Term | 12 | 8 | 2.00 | - |
| Term | 12 | 9 | 2.10 | - |
| Term | 12 | 10 | 2.20 | - |
| Term | 12 | 11 | 2.30 | - |
| Term | 12 | 12 | 2.40 | - |
| Term | 12 | 13 | 2.60 | - |
| Term | 12 | 14 | 2.62 | - |
| Term | 12 | 15 | 2.80 | - |
| Term | 12 | 16 | 2.90 | - |
| Term | 12 | 17 | 3.08 | - |
