# Supplementary material for: Does birth weight affect neonatal body weight, growth, and physiology in an animal model?
Source: PLoS One. 2021 Feb 16;16(2):e0246954. doi: 10.1371/journal.pone.0246954 (PMC7886147; doi:10.1371/journal.pone.0246954)
Supplement: S2 Table — Measurements of different swallowing behaviors made on both preterm and term individuals at different days of the postnatal period. Litter/Sow identification is also listed to indicate whether the litter number and term status. (DOCX) [file pone.0246954.s002.docx]

**S2 Table. Swallowing behavior measurements for term and preterm infants.**

| **Birth Status** | **Age (days)** | **Individual** | **Birth weight (kg)** | **Swallow Duration (sec)** | **Sucks/Swallow** | **Swallow Delay (percentage)** | **Litter** |
| --- | --- | --- | --- | --- | --- | --- | --- |
| Preterm | 7 | 1 | 0.52 | 0.02 | 3.00 | 53.12 | PT1 |
| Preterm | 7 | 3 | 0.70 | 0.02 | 2.83 | 53.38 | PT1 |
| Preterm | 7 | 4 | 0.59 | 0.03 | 2.77 | 43.30 | PT1 |
| Preterm | 7 | 6 | 0.88 | 0.03 | 2.50 | 19.40 | PT1 |
| Preterm | 7 | 7 | 0.30 | 0.02 | 3.00 | 25.28 | PT1 |
| Preterm | 7 | 13 | 1.26 | 0.10 | 4.33 | 47.43 | PT2 |
| Preterm | 7 | 14 | 1.29 | 0.10 | 2.29 | 38.30 | PT3 |
| Preterm | 7 | 15 | 0.71 | 0.14 | 4.35 | 48.74 | PT3 |
| Preterm | 7 | 16 | 1.03 | 0.10 | 2.84 | 46.94 | PT3 |
| Preterm | 7 | 17 | 0.94 | 0.12 | 2.95 | 45.83 | PT3 |
| Preterm | 17 | 1 | 0.52 | 0.02 | 3.58 | 21.33 | PT1 |
| Preterm | 17 | 3 | 0.70 | 0.02 | 2.16 | 19.91 | PT1 |
| Preterm | 17 | 4 | 0.59 | 0.02 | 2.37 | 23.99 | PT1 |
| Preterm | 17 | 6 | 0.88 | 0.02 | 2.26 | 22.94 | PT1 |
| Preterm | 17 | 7 | 0.30 | 0.02 | 2.90 | 22.85 | PT1 |
| Preterm | 17 | 13 | 1.26 | 0.04 | 1.23 | 19.64 | PT2 |
| Preterm | 17 | 14 | 1.29 | 0.07 | 1.17 | 26.83 | PT3 |
| Preterm | 17 | 15 | 0.71 | 0.08 | 0.68 | 26.35 | PT3 |
| Preterm | 17 | 16 | 1.03 | 0.07 | 2.06 | 24.83 | PT3 |
| Preterm | 17 | 17 | 0.94 | 0.07 | 0.58 | 25.39 | PT3 |
| Term | 7 | 8 | 1.25 | 0.02 | 2.19 | 24.34 | T1 |
| Term | 7 | 10 | 1.32 | 0.02 | 2.45 | 16.37 | T1 |
| Term | 7 | 11 | 1.70 | 0.02 | 1.68 | 33.85 | T1 |
| Term | 7 | 18 | 0.80 | 0.06 | 2.05 | 26.96 | T2 |
| Term | 7 | 19 | 1.50 | 0.07 | 1.90 | 31.28 | T2 |
| Term | 7 | 20 | 1.50 | 0.06 | 1.67 | 23.57 | T2 |
| Term | 7 | 21 | 1.00 | 0.06 | 2.19 | 24.77 | T2 |
| Term | 7 | 22 | 0.95 | 0.06 | 0.45 | 22.62 | T3 |
| Term | 7 | 23 | 1.03 | 0.07 | 1.80 | 24.47 | T3 |
| Term | 7 | 24 | 0.89 | 0.06 | 3.50 | 18.83 | T3 |
| Term | 7 | 25 | 1.00 | 0.07 | 0.25 | 23.89 | T3 |
| Term | 17 | 8 | 1.25 | 0.03 | 1.80 | 12.41 | T1 |
| Term | 17 | 10 | 1.32 | 0.02 | 2.00 | 13.49 | T1 |
| Term | 17 | 11 | 1.70 | 0.03 | 2.00 | 11.71 | T1 |
| Term | 17 | 18 | 0.80 | 0.05 | 1.00 | 13.65 | T2 |
| Term | 17 | 20 | 1.50 | 0.03 | 1.00 | 10.57 | T2 |
| Term | 17 | 21 | 1.50 | 0.03 | 0.65 | 1.55 | T2 |
| Term | 17 | 22 | 1.00 | 0.03 | 1.83 | 13.35 | T2 |
| Term | 17 | 27 | 0.95 | 0.06 | 2.05 | 22.47 | T3 |
| Term | 17 | 23 | 1.03 | 0.07 | 3.00 | 23.22 | T3 |
| Term | 17 | 24 | 0.89 | 0.05 | 1.11 | 21.04 | T3 |
| Term | 17 | 25 | 1.00 | 0.06 | 0.90 | 19.56 | T3 |
